# Essential updates 2018/2019: Liver transplantation

**DOI:** 10.1002/ags3.12321

**Published:** 2020-02-25

**Authors:** Masahiro Ohira, Naoki Tanimine, Tsuyoshi Kobayashi, Hideki Ohdan

**Affiliations:** ^1^ Department of Gastroenterological and Transplant Surgery Graduate School of Biomedical and Health Sciences Hiroshima University Hiroshima Japan; ^2^ Medical Center for Translational and Clinical Research Hiroshima University Hospital Hiroshima Japan

**Keywords:** hepatitis C virus, hepatocellular carcinoma, immunosuppression, liver graft, liver transplantation

## Abstract

Among the recent topics in the field of liver transplantation (LT), one of the significant therapeutic breakthroughs is the introduction of direct‐acting antiviral agents (DAAs) against hepatitis C virus (HCV) infection. With cure rates close to 100%, a better proportion of LT candidates and recipients can be cured of HCV infection by DAA therapies that are simple and well‐tolerated. Other critical topics include the issue of indication of LT for patients with hepatocellular carcinoma, which has been continuously studied. Several expanded criteria beyond the Milan criteria with acceptable results have been recently reported. The role of donor‐specific antibodies (DSAs) in intractable rejection is also an important matter that has been studied. Although long recognized as an important factor in antibody‐mediated rejection and even graft survival in renal transplantation, the impact of DSAs on graft and patient survival in LT remains to be elucidated. Including the issues described above, this article focuses on recent advances in LT, management to avoid recurrence of primary diseases, optimization of immunosuppressive treatment, and extended donor criteria.

## 1 INTRODUCTION

Liver transplantation (LT) is a prevalent treatment option for end‐stage liver disease and acute liver failure, although many characteristic issues remain to be solved. Favorable outcomes require careful screening for eligible recipients, proper selection of well‐matched live or cadaveric donors at the appropriate time, optimization of immunosuppressive treatment, and preemptive and/or therapeutic treatment to avoid rejection and recurrence of primary diseases including hepatocellular carcinoma (HCC). Focusing on those issues, this article summarizes the recent advances in LT.

## 2 HEPATOCELLULAR CARCINOMA

### 2.1 Predictors and criteria

Since the Milan criteria was created for the eligibility of LT in patients with HCC,^1^ many studies were published aiming to expand the Milan criteria without impairing patient survival or recurrence‐free survival.^2^, ^3^, ^4^, ^5^ The most important articles aiming at defining predictors or criteria for LT patients with HCC published in the last 2 years between 2018 and 2019 are summarized in Table 1.^6^, ^7^, ^8^, ^9^, ^10^, ^11^, ^12^, ^13^, ^14^ In Japan, Shimamura et al^6^ recently proposed a new criterion—the 5‐5‐500 rule—which is the expanded living‐donor liver transplantation (LDLT) criteria for HCC patients based on a retrospective study of Japanese nationwide survey. They demonstrated that the HCC patients within the 5‐5‐500 rule had 7.3% of recurrence at 5 years after LT. Mazzaferro et al^7^ developed a prognostic model named Metroticket 2.0 Model which predicted a 70% chance of HCC‐specific survival 5 years after LT according to tumor size, numbers, and alpha‐fetoprotein (AFP) value at LT. They showed that the 5‐year HCC‐specific survival was significantly better in patients within the Metroticket 2.0 Model compared with those without the Model (90.1% vs 66.6%; *P < *.001). Hazard Associated with Liver Transplantation for Hepatocellular Carcinoma (HALTHCC) model has been recently validated by international multicenters including Japanese centers.^8^ This model is a continuous score calculated as follows: (2.31*lin(AFP)) + (1.33*tumor burden score) + (0.25*MELD‐Na) − (5.57*Asia). Five‐year post‐LT HCC recurrence ranged from 8.6% (HALTHCC < 5; n = 145 in 3068) to 70.0% (HALTHCC > 35; n = 24 in 3068). HALTHCC score predicted the vascular invasion and poorly differentiated component on explant pathology. Mehta et al^15^ have developed Validation of a Risk Estimation of Tumor Recurrence After Transplant (RETREAT) Score including three variables that independently predicted post‐LT HCC recurrence: AFP at LT, microvascular invasion, and the sum of the largest viable tumor diameter and the number of viable tumors on explant. The authors recently validated RETREAT score by United Network for Organ Sharing (UNOS) between 2012 and 2014. Overall HCC recurrence was found in 4.4% (145/3276). Post LT HCC recurrence probability at 3 years was 1.6% of patients with a RETREAT score of 0, 8.4% with a score of 3, and 29.0% for a score of 5 or higher (*P* < .001).^9^ RETREAT score should be used for standardizing post‐LT HCC surveillance strategies.

**Table 1:** Prediction of HCC recurrence after liver transplantation published in the 2‐y period between 2018 and 2019

Author	Year	Patient Number	Information
Shimamura^6^	2019	965	The 5‐5‐500 rule (nodule size ≤5 cm in diameter, nodule number ≤5, and AFP value ≤500 ng/mL): 5‐y recurrence rate of 7.3% and a 19% increase number in the eligible patients who are beyond Milan criteria
Mazzaferro^7^	2018	1018 in training set, 341 in validation set	For patients with HCC to have a 70% chance of HCC‐specific survival 5 y after transplantation, their level of AFP should be <200 ng/mL and the sum of number and size of tumors should not exceed 7; if the level of AFP was 200‐400 ng/mL, should be ≤5; if their level of AFP was 400‐1000 ng/mL, should be ≤4. In the validation set, the model identified patients who survived 5 y after liver transplantation with 0.721 accuracy
Firl 8	2019	4089	The Hazard Associated with Liver Transplantation for Hepatocellular Carcinoma (HALTHCC) model is a continuous score calculated as follows; (2.31*lin(AFP)) + (1.33*tumor burden score) + (0.25*MELD‐Na) − (5.57*Asia). HALTHCC score predicted overall survival, recurrence rate, and vascular invasion, poorly differentiated components on explant pathology
Mehta^9^	2018	3276	RETREAT score predicts post LT HCC recurrence. Post‐LT survival at 3 y; 91% for a score 0, 80% for a score of 3, and 58% for a score ≥5 (*P* < .001). Post‐LT HCC recurrence probability within 3 y increased from 1.6% with RETREAT score of 0 to 29% for a score ≥5 (*P* < .001)
Lee^10^	2018	328	After propensity score matching, 82 patients with GRWR <0.8% and 246 patients with GRWR ≥0.8%. For patients with HCC beyond Milan criteria, 1‐, 3‐, and 5‐y recurrence‐free survival rates were 52.4%, 49.3%, and 49.3%, respectively, for patients with GRWR <0.8%, and 76.5%, 68.3%, and 64.3%, respectively, for patients with GRWR ≥0.8%; *P* = .049
Meischl^11^	2019	216	CRP >1 mg/dL was an independent risk factor for HCC recurrence with a 5‐y recurrence rate of 27.4% vs 16.4%. OS was similar in patients with normal vs elevated CRP levels
Kornberg^12^	2019	123	ALBI grade calculated using pre‐LT serum albumin and bilirubin. Posttransplant HCC recurrence rates were 10.5%, 15.9%, and 68.2% in ALBI grade 1, 2, and 3, respectively. Along with AFP and CRP, ALBI grades 1 or 2 was identified as an independent predictor of RFS. ALBI grade 3 proved to be the strongest indicator of microvascular invasion
Mano^13^	2018	216	A low Lymphocyte‐to‐Monocyte Ratio (LMR) was associated with poor prognosis and represented an independent prognostic factor, particularly among patients beyond Milan criteria. The ratio of CD3‐positive to CD68‐positive cells was significantly lower in the low‐LMR group
Silva^14^	2018	15 043	A history of prior upper abdominal surgery was associated with an increased risk of graft survival and overall survival after LT for HCC

Abbreviations: AFP, alpha‐fetoprotein; ALBI grade, albumin‐bilirubin score (formula: 0.66 × log10 [total bilirubin μmol/L] – 0.085 × [albumin g/L]), classified as grade 1 (≤−2.60), grade 2 (−2.60 to −1.39), or grade 3 (>−1.39), respectively; CRP, c reactive protein; GRWR, graft to recipient weight ratio; HCC, hepatocellular carcinoma; LT, liver transplantation; MELD, Model For End‐Stage Liver Disease; OS, overall survival.

Small‐for‐size grafts defined as GRWR <0.8% showed poor oncologic outcomes, including recurrence‐free survival and overall survival, among the patients with HCC, beyond the Milan criteria.^10^ It has been speculated that one of the possible mechanisms can be that high portal pressure and liver congestion affects inflammation of endothelial cells and tumor immunity in the liver. Another interesting finding would be that a history of prior upper abdominal surgery decreased graft survival and overall survival after LT for HCC, although no convincing mechanism has been elucidated.^14^ Besides, several serum biomarkers, including CRP,^11^ ALBI grade,^12^ and LMR^13^ have been recently published.

### 2.2 Downstaging

Downstaging would be a potential option for patients with advanced HCC to undergo LT as a curative treatment. The American Association for the Study of Liver Disease (AASLD) guidelines recommended that patients beyond the Milan criteria (T3) should be considered for LT after successful downstaging into the Milan criteria.^16^ The problem is that the inclusion criteria and downstaging protocols are not systematically fixed.^17^ Essential and the last 2‐year updates of downstaging HCC before LT are summarized in Table 2.^18^, ^19^, ^20^, ^21^, ^22^, ^23^

**Table 2:** Downstaging for advanced HCC prior to liver transplantation published in the 2‐y period between 2018 and 2019

Author	Year	Patient Number	Information
Pommergaard^18^	2018	4978 LRT of 23 124 LT recipients with HCC	Locoregional therapy was associated with improved OS (HR 0.84 [0.73‐0.96]) and HCC‐specific survival (HR 0.76 [0.59‐0.98]) after LT. RFA was highly beneficial for OS and HCC‐specific survival after LT
Ogawa^19^	2019	223 LT recipients with HCC	Regarding the number of pretreatments, recurrence rate was significantly higher in the ≥5 pretreatments group than the 0 group. However, for patients meeting Kyoto criteria, there were no significant differences in recurrence rates between groups
Mehta^20^	2019	407 HCC LT recipients with >1000 ng/mL of AFP at waiting list	5‐y OS: AFP >1000 at LT; 48.8%, AFP to 101‐499; 67.0%, AFP to <100; 88.4% 5‐y HCC recurrent probability: AFP >1000; 35.0%, AFP to 101‐499; 13.3%, AFP to <100; 7.2% In multivariate analysis; a decrease in the AFP to 101‐499 was associated with a >2‐fold reduction in post‐transplant mortality (*P* = .01) and a nearly 3‐fold reduction in HCC recurrence (*P* = .02)
Sinha^21^	2019	UNOS database of 3819 HCC LT; always within Milan (n = 3276), UNOS‐DS (n = 422), and AC‐DS (n = 121)	On explant, vascular invasion was found in 23.7% of AC‐DS versus 16.9% of UNOS‐DS and 14.4% of Milan (*P* = .002). Within down‐staging groups, risk of post‐LT death was increased in SWR (short wait regions) or MWR (mild wait regions) and with AFP >100 ng/mL at LT. The 3‐y HCC recurrence probability was 6.9% for Milan, 12.8% for UNOS‐DS, and 16.7% for AC‐DS (*P* < .001). In down‐staging groups, AFP >100 was the only independent predictor of HCC recurrence
Vutien^22^	2019	16 558 HCC patients underwent LT in SRTR data	HCC burden measured at 3 points on the initial waiting list (I), maximum (M) total tumor diameter, and last (L) exception petition. Classification; (A) <Milan (B) Milan (C) >Milan to UCSF (D) >UCSF. 1233 (7%) had any post‐LT rHCC. rHCC rates were higher in RH‐IML group CCC (15%) DDD (18%). Low recurrence rates: M and L tumor burden did not exceed Milan (class A or B), successful down staging when L was A(<Milan) and M tumor burden did not exceed I, as in BBA, CCA, and DDA
DiNorcia^23^	2019	4109 patients for validation between 2015 and 2017"	Compared with patients without cPR, cPR patients were younger; had lower MELD scores, AFP levels, and NLR; were more likely to have tumors within Milan criteria and fewer LRT treatments; and had significantly lower 1‐, 3‐, and 5‐y incidence of post‐LT recurrence (1.3%, 3.5%, and 5.2% vs 6.2%, 13.5%, and 16.4%; *P* < .001) and superior overall survival (92%, 84%, and 75% vs 90%, 78%, and 68%; *P* < .001). Multivariable predictors of cPR included age, sex, liver disease diagnosis, MELD, AFP, NLR, radiographic Milan status, and number of LRT treatments

Abbreviations: AC‐DS, all‐comers downstaging; AFP, alpha‐fetoprotein; cPR, complete pathological response; HCC, hepatocellular carcinoma; HR, hazard ratio; Kyoto criteria, tumor number ≤10, maximal diameter of each tumor ≤5 cm, and serum des‐gamma‐carboxy prothrombin (DCP) levels of ≤400 mAU/mL; LRT, Locoregional therapy; LT, liver transplantation; MELD, Model For End‐Stage Liver Disease; NLR, neutrophil lymphocyte ratio; OS, overall survival; RFA, radiofrequency ablation; rHCC, recurrent HCC; UCSF criteria, 1 tumor >5 cm and up to 6.5 cm or 3 tumors each up to 4.5 cm; UNOS‐DS, (one lesion >5 cm and ≤8 cm; two to three lesions each ≤5 cm; or four to five lesions each ≤3 cm with total tumor diameter ≤8 cm) downstaging.

A recent retrospective cohort of 23 124 LT patients with HCC registered in the European Liver Transplant Registry database revealed that the locoregional treatment (4978 of 23 124) while on a waiting list was associated with improved OS (HR 0.84 [0.73‐0.96]) and HCC‐specific survival (HR 0.76 [0.59‐0.98]) after LT.^18^ RFA was highly beneficial for OS and HCC‐specific survival after adjusting for related factors. On the other hand, the Kyoto group recently reported that any pretreatment significantly increased the recurrence rate after LT compared with no pretreatment.^19^ However, for patients meeting the Kyoto criteria, they found that there were no significant differences in recurrence rates between groups.

The effect of locoregional therapy (LRT) should be important for LT patients with advanced HCC. UCSF group analyzed LT patients with HCC who had at least one AFP value >1000 ng/mL while on the LT waiting list in the UNOS database. A reduction rate of AFP was significantly correlated with posttransplant mortality or HCC recurrence rates after LT.^20^ The same group clarified the efficacy of USCF downstaging criteria compared with patients who had an initial tumor burden exceeding USCF criteria.^21^ Vutien et al^22^ advocated a useful tool for evaluating risk for recurrent HCC after LT by classifying HCC burden as Milan and UCSF criteria at initial, maximum, and last exception petition. Regarding pathological responses after LRT, complete pathological responses were significantly associated with lower post‐transplant recurrence and superior survival (5.2% vs 16% at 5‐year; *P* < .001).^23^ The predictive factors for complete pathological responses were identified several non‐tumor‐related factors including age, sex, and MELD score at LT in addition to tumor‐related factors such as size, number, NLR, and AFP.

### 2.3 Post LT management: immunosuppression and adjuvant therapy

There is no standard management including immunosuppression and adjuvant therapy for LT with HCC after LT. Mechanistic targets of rapamycin (mTOR) inhibitors have antiangiogenic and antiproliferative effects in several experimental models.^24^, ^25^ A recent randomized trial could not provide evidence for the preventive effect of mTOR inhibitors on preventing HCC recurrence after LT.^26^ On the other hand, the recent meta‐analysis of 23 studies including 17 observational and six randomized trials demonstrated that recurrence‐free survival and recurrence rate in the mTOR inhibitor group were improved compared with CNI control groups.^27^ Mycophenolic acid (MPA) and its prodrug, mycophenolate mofetil (MMF), are generally used after LT. MPA was also reported to have an antiproliferative effect in several cancer models.^28^, ^29^, ^30^ Chen et al^31^ recently demonstrated the antitumor effect of MPA by human cell lines and the organoids model. Among 44 LT recipients with HCC, the use of MMF had a lower risk of HCC recurrence and improved overall survival.

Immunotherapy for HCC targeting immune checkpoints is currently underway in several clinical studies and shows potential in HCC treatment.^32^ However, immune checkpoint inhibitors are concerned about breaking the balance of immune tolerance in organ transplant patients. In fact, immune checkpoint inhibitors could cause sometimes fatal organ rejection.^33^, ^34^

## 3 HEPATITIS B VIRUS/HEPATITIS C VIRUS

From 2018 to 2019, there is no appreciable clinical advance in the management of the hepatitis B virus (HBV) in the LT field. In contrast, the management of patients with hepatitis C virus (HCV) infection has been dramatically changing after the emergence of direct‐acting antivirals (DAAs). Due to the high efficacy for obtaining sustained virologic response (SVR) and improvement of liver function, even for the patients with cirrhosis, a major decline was observed in the number of LT performed both in patients with decompensated cirrhosis with HCV and in those with hepatocellular carcinoma associated with HCV in the United States^35^ and European countries.^36^, ^37^ Furthermore, the survival of LT recipients with HCV‐related liver disease has clearly improved because of treatment for HCV recurrence, which is a common problem in clinical practice.^36^, ^38^ Six studies have reported the efficacy of DAA combination regime for post‐LT setting from 2018 to 2019 (Table 3).^39^, ^40^, ^41^, ^42^, ^43^, ^44^

**Table 3:** Study with DAAs based therapy reported during 2018‐2019

Regimen	Type of study	Target HCV genotype	Transplantation	Number of patients	Treatment duration	12 SVR achievement
Sofosbuvir/velpatasvir^39^	Phase II, open‐label study	1,2,3,4	LT	79	12 wk	96.0%
Sofosbuvir/NS5Ai ± rivabirin^40^	Retrospective study	1,3	LT	78	12 (24) wk	89.4%
Sofosbuvir/NS5Ai ± rivabirin^41^	Prospective multicenter study	1,2,3,4,5	LT	512	12 (24) wk	96.1%
Ombitasvir/paritaprevir/ritonavir + dasabuvir + rivabirin^42^	Retrospective study	1	LT	127	12 (24) wk	81.1%^a^
Glecaprevir/pibretasvir^43^	Phase III, open‐label study	1,2,3,4,5,6	KT/LT	20/80	12 wk	98.1%
Glecaprevir/pibretasvir^44^	Prospective multicenter study	1,2	LT	25	8 (12) wk	96.0%

Abbreviations: DAA, direct antiviral agent; KT, kidney transplantation; LT, liver transplantation; NS5Ai, nonstructural protein 5A inhibitor; SVR, sustained.

a All the patients had previous history of failure with boceprevir or telaprevir based therapy virus response.

Along with these successful DAA treatments, a couple of considerations were reported. Erard et al^45^ reported a case of late relapse of HCV infection followed by LT after 2 years of virologic response with DAA therapy. This report suggests the requirement of HCV RNA monitoring to detect HCV relapse post LT even after a long virologic response. Reactivation of HBV infection during DAA therapy for HCV occurs in patients with chronic HBV and HCV co‐infection within the early phase (4‐8 weeks) of DAA treatment.^46^ However, Vionnet et al^47^ reported a year‐late HBV reactivation following DAA therapy for recurrent HCV infection post LT. This report suggests that reactivation  of latent HBV can take for significant time and long‐time monitoring of HBV DNA is recommended after DAA therapy in post LT setting.

Additionally, the high efficacy of DAA treatment offered another consideration for the management of the patients on the waiting list for LT. The improvement of liver functions don't always reach the point of delisting and/or improvement of complications from portal hypertension such as encephalopathy or intractable ascites. The patient with improved MELD score decreases the probability of receiving an LT under the MELD allocation systems (the so‐called MELD purgatory).^48^ The debate about the optimal timing for treating HCV patients in LT waiting list is ongoing, and this issue very much depends on the medical environment, such as whether a patient can receive organ donation.

Along with the development of antiviral therapy, including HBV and HCV, the idea of transplanting an organ from viral positive donors to negative recipients is spreading to overcome organ shortage. The topic of viral exposed organs will discuss in following donor section.

## 4 ACUTE HEPATITIS

Liver transplantation is a vital treatment for patients with acute liver failure. Patients diagnosed with acute liver failure categorized as status 1 on the MELD‐based allocation system received the highest priority on the waiting list. Recent studies focus on another cohort with acute‐on‐chronic liver failure (ACLF), which identifies patients with chronic liver disease who develop sudden exacerbation of liver function and high short‐term mortality. Sundaram et al^49^ showed high mortality with ACLF based on European Association for the Study of the Liver (EASL) criteria (Table 4) with three or more failing organs (ACLF‐3) on the LT waiting list, even among those with lower MLED‐Na scores, and LT clearly improved survival for these patients. Thuluvath et al also reported the probability of staying alive >30 days on the waitlist without LT was inversely related to the number of organ failure on ACLF and the probability of patients with ACLF‐3 was only 2%–8%.^50^ Together with the dramatic improvement of 1‐year survival with LT (around 80%), even with the marginal organ, these findings illustrate the need for prioritizing these patients. There are other prominent ACLF criteria by the Asian Pacific Association for the Study of the Liver (APASL) which focus more on liver function than EASL (Table 4). Mahmud et al showed the discordance of ACLF between APASL and EASL, and ACLF that met both definitions had poor survival compared to those with APASL or EASL ACLF alone.^51^, ^52^ These data should be considered as an account of the liver allocation system with local organ supply.

**Table 4:** Definition of acute‐on‐chronic liver failure

European Association for the Study of the Liver (EASL)	Asia‐Pacific Association for the Study of the Liver (APASL)
Acute deterioration of preexisting, chronic liver disease, usually related to a precipitating event and associated with increased mortality at 3 mo due to multisystem organ failure (following definitions). Organ failure definitions Liver failure: serum bilirubin ≥12.0 mg/dL Kidney failure: serum creatinine ≥2.0 mg/dL or the use of renal replacement therapy Cerebral failure: grade III or IV hepatic encephalopathy, according to the West Haven classification Coagulation failure: INR >2.5 and/or a platelet count of 20 × 10^9^/L Circulatory failure: use of dopamine, dobutamine, or terlipressin Respiratory failure: PaO_2_/FiO_2_ ≤200 or an SpO_2_/FiO_2_ ≤200.	Acute hepatic insult manifesting as Jaundice (serum bilirubin ≥5 mg/dL (85 μmol/L) and coagulopathy (INR ≥ 1.5 or prothrombin activity <40%) Complicated within 4 wk by clinical ascites and/or encephalopathy Patient with previously diagnosed or undiagnosed chronic liver disease/cirrhosis, and is associated with a high 28‐d mortality

## 5 NON‐ALCOHOLIC STEATOHEPATITIS/ALCOHOLIC

### 5.1 Non‐alcoholic steatohepatitis (NASH)

Owing to the improvement of HCV management, non‐alcoholic fatty liver (NAFLD)/non‐alcoholic steatohepatitis (NASH) became a leading indication of LT in the United States and European countries.^53^, ^54^ Younossi et al^54^ showed NASH is the most rapidly growing cause of HCC in the American database, the Scientific Registry of Transplant Recipients. In spite of specific patient's background, that is, higher comorbidities of metabolic diseases, the outcome of LT for NASH are likely comparable with other disease indication with HCC^55^ or without HCC.^56^ Post‐LT is a high risk for metabolic diseases including diabetes, hypertension, hyperlipidemia, and also NAFLD/NASH because of the mandatory use of immunosuppressants. Although post‐LT NAFLD/NASH shows slow progression, and only a small population develop cirrhosis again, NASH related LT recipients have been reported as a higher risk for quick deterioration for fibrosis and close management may be required.^57^ Based on the current growing trend of this topic, several excellent reviews about etiology and management are available elsewhere.^54^, ^58^, ^59^

### 5.2 Alcoholic

Alcohol‐related liver disease (ALD) is also becoming a leading indication for LT in Western countries. Most organ allocation policies set some period (6‐18 months) of abstinence to discriminate whether liver function could improve to avoid LT. However, this period certainly reflects ethical and social ideas based on the deeply ingrained view of alcohol‐use disorder as simply self‐inflicted behavior. This period is often an unrealistic barrier because the prognosis of patients with acute onset alcoholic hepatitis shows 75%–90% mortality within 2 months when they are not responsive for medical therapy except LT. Lee et al^60^ showed the selective use of LT can be a lifesaving option for refractory alcoholic hepatitis (AH), and the 3‐year survival rate and frequency of alcohol use after transplantation appear to be accepted without any abstinence period by retrospective analysis of 147 American cases. They also showed the early LT for selected severe AH increases survival periods of patients, regardless of estimated risk of sustained alcohol use after LT with a mathematical model.^61^ This is a consistent result shown in a landmark study of this issue with a small cohort (n = 26) from France.^62^ Together with the idea of heterogeneous genetic predisposition to ALD,^63^, ^64^ the time is coming to reconsider the indication of LT for ALD patients.

## 6 PRIMARY BILIARY CHOLANGITIS/PRIMARY SCLEROSING CHOLANGITIS

### 6.1 Primary biliary cholangitis (PBC)

The proportion of PBC decreased from 20% of LT in 1986 to 4% in 2015, as per the European Liver Transplantation Registry.^65^ However, LT remains the only radical treatment for PBC. Although the prognosis of PBC after LT is relatively good, the recurrence rates of PBC were in a range from 9% to 35% after LT.^66^ Risk factors for recurrent PBC were reported as recipient/donor age, recipient/donor gender, recipient HLA status, ischemic time, and immunosuppression.^66^ Recent multicenter study (785 patients with PBC who underwent LT) from North America and Europe reported that younger age, use of tacrolimus, and liver dysfunction early after LT were associated with an increased risk of PBC recurrence.^67^ The authors advocated that cyclosporine might have a protective effect on the recurrence of PBC; however, the opposite result was that cyclosporine was a risk factor of PBC recurrence in a Japanese multicenter study.^68^ Early use of UDCA after LT was associated with reduced risk of recurrence of PBC.^69^ Some clinical trials for PBC treatments including obeticholic acid^70^ or bezafibrate^71^, ^72^ need to be investigated in LT patients whether to decrease the risk of recurrent PBC after LT.

### 6.2 Primary sclerosing cholangitis (PSC)

A Japanese multicenter study of LDLT for primary sclerosing cholangitis (PSC) revealed that MELD score, first‐degree relative donors, CMV infection, and early biliary anastomotic complications were risk factors for recurrence of PSC after LT.^73^ A recent systematic review including 2159 patients who underwent LT for PSC revealed that cholangiocarcinoma before LT, inflammatory bowel disease, older donor age, higher MELD score, and acute cellular rejection increased the risk of recurrent PSC. It was also showed that colectomy before LT reduced the risk of recurrent PSC.^74^ Another systematic review concluded that the data favored a protective role of colectomy in recurrent PSC but the current evidence was not strong enough to recommend routine colectomy for recurrent PSC prevention.^75^ Andres et al^76^ presented a new calculator that accurately estimated individual post LT survival for PSC patients. The calculator is available at: http://pssp.srv.ualberta.ca/calculator/liver_transplant_2002. Inflammatory bowel disease (IBD) associated with PSC has a higher risk of colorectal cancer than other forms of IBD. However, there are no definite treatment strategies for IBD yet.^77^ A French multicenter study revealed that anti‐TNF therapy including infliximab or adalimumab following LT for PSC yielded 67% of clinical response and 39% of clinical remission among 18 patients recruited from nine LT centers in France.^78^

## 7 DSA AND ABO INCOMPATIBILITY

### 7.1 Impact of anti‐human leukocyte antigen donor‐specific alloantibodies (DSAs) developing after liver transplantation

The incidence and impact of anti‐human leukocyte antigen donor‐specific alloantibodies (DSAs) developing after LT remains controversial and not extensively studied. University Medical Center Hamburg‐Eppendorf group in Germany retrospectively investigated the role of preformed DSA in long‐term liver allograft survival by analyzing 177 pre‐transplant sera of first LT patients. They defined a MFI of >1500 as positive with Luminex single antigen technology.^79^ They found that acute rejections or ischemic‐type bile duct lesions (ITBL) were not higher in the DSA group, and there was no difference in long term graft function or survival in patients without HLA‐Ab, with non‐DSA, or with DSA. The French single‐center retrospective study demonstrated that patient survival and graft survival were not significantly different according to the presence or not of de novo DSAs at 1 year, although acute rejection and portal fibrosis were more frequent at 1 year for patients with DSAs.^80^ Contrary to those results suggesting that DSAs have limited overall impact on graft and patient outcome in LT patients, Hannover Medical School group investigating gene expression of various transcripts in biopsies of liver allografts revealed that a humoral allo‐sensitization as indicated by the appearance of DSA was associated with more subclinical graft injury, more graft fibrosis, and upregulation of clinical T‐cell‐mediated rejection (TCMR) associated transcripts.^81^ Another group in the United State demonstrated that a prominent IgG4 DSA profile was strongly correlated with greater HLA mismatch, a histopathological phenotype characterized by the presence of interface activity with variable degrees of fibrosis, and a transcriptional profile of attenuated TCMR.^82^ Hence, the appearance or persistence of DSA in the context of TCMR may prompt closer monitoring and reevaluation of the immunosuppressive regimen in those patients.

Immunogenic HLA regions, known as epitopes, are composed of polymorphic sequences of amino acid residues termed eplets. There is a tendency to suggest that epitope matching is likely to be superior to broad antigen HLA matching such that the allocation of donor organs to patients with a more favorable epitope compatibility profile may lead to better allograft outcomes. Indiana University group demonstrated that donor‐recipient HLA epitope mismatch was significantly associated with a risk of de novo DSA formation and rejection after LT.^83^

### 7.2 ABO incompatibility

ABO‐incompatible (ABO‐i) LT is an inevitable option in the point of view of the limited donor pool for LDLT; however, naturally occurring antibodies (Abs) against blood group A or B (A/B) carbohydrate determinants in sera are a major impediment to achieving successful transplantation. Rituximab has greatly improved the outcomes of ABO‐i LDLT. A Japanese multicenter study group established the efficacy and safety of rituximab in adult patients undergoing ABO‐i LDLT, although an optimal regimen had not been defined.^84^ Rituximab had been used in combination with other desensitization treatment regimens, like pretransplant immunosuppressive drugs, pretransplant plasmapheresis, posttransplant local infusion therapy, and splenectomy. The recent retrospective study demonstrated that pretransplant rituximab without additional treatments yielded satisfactory outcomes comparable to that with additional treatments, such as plasmapheresis and local infusion therapy.^85^

Despite the acceptable outcome of ABO‐i LDLT brought by the introduction of rituximab, there is a paper concerned about the impact of ABO‐i transplants on liver graft regeneration.^86^ This retrospective study showed that the absolute liver graft volumes at 3 weeks after LT were significantly lower in the ABO‐i LT patients than those in the ABO compatible (ABO‐c) patients in the propensity score‐matched patients. Hence, graft regeneration may need to be intensively investigated using a volumetric assessment in patients who have undergone ABO‐i LDLT.

In addition to studies evaluating the outcomes of ABO‐i LDLT, studies have been undertaken on immunological concerns about the desensitization protocol for breaking through the ABO‐i barrier and whether it could have a positive or negative impact on the host immune status. A Japanese national survey demonstrated that the ABO‐i LDLT recipients with PSC, who had been treated with rituximab, persistently retained an excellent graft function without any recurrence of PSC.^87^ This study presented what they suggested to be a novel paradigm for preventing the recurrence of PSC, which frequently recurs after ABO‐c LDLT. Several other studies were focused on the immunological concerns that the desensitization for ABO‐i LT might have a negative impact on the recurrence of hepatocellular carcinoma (HCC), which, with severe liver cirrhosis, is the common indication for LT.^88^, ^89^, ^90^ All of those studies demonstrated no significant differences in the long‐term overall survival and recurrence‐free survival rates between patients receiving ABO‐c or ABO‐I LDLT. Hence, ABO‐i LDLT constitutes a potentially feasible option for patients with HCC, especially those with compensated cirrhosis with HCC within conventional Milan criteria.

## 8 LIVER TRANSPLANTATION AND IMMUNE TOLERANCE

The liver exhibits intrinsic immune tolerogenic properties that contribute to a unique propensity toward spontaneous acceptance when transplanted, both in animal models and even in human clinical settings. Unlike transplantation of other solid organs, for several years following LT a non‐negligible subset of patients is capable of maintaining normal allograft function without any immunosuppressive drug treatment. Significant efforts have been made to identify sensitive and specific biomarkers of immune‐tolerance in order to stratify LT recipients according to their need for immunosuppressive medication and their likelihood of being able to completely discontinue it.

A prospective pilot study measuring immune markers, including the ratio of regulatory T (Treg) and T helper (Th) 17 cells in peripheral blood of LT recipients revealed that the Treg/Th17, Th1/Th17, and CD8/Th17 ratio in tolerant recipients was significantly increased compared with that of nontolerant recipients.^91^ This result suggests that Treg cells play an essential role in inducing and keeping immune tolerance in LT. From among all the Treg cell mechanisms related to their suppressive capacity, adenosine triphosphate (ATP) metabolism is one that is well documented. In this context, there are essential players that constitute the CD39/CD73 axis. A Spanish group investigated the action of extracellular nucleotides in human T cells to examine the influence of CD39/CD73 ectonucleotidases and subsequent adenosine signaling through adenosine 2 receptor in the induction of clinical tolerance after LT.^92^ They found that the expression of the enzyme responsible for the degradation of adenosine, adenosine deaminase, was higher in tolerant patients with respect to the nontolerant group along the immunosuppression withdrawal, suggesting that extracellular adenosine signaling and its degradation by the sequential action of CD39 and CD73 plays a role in the complex system of regulation of LT tolerance.

It is well known that the susceptibility of inducing immune tolerance depends on the type of immunosuppressant used after LT. A key difference between the mammalian target of rapamycin inhibitor (mTOR‐I) and calcineurin inhibitor (CNI) is their effect on Tregs (CD4^+^CD25highFoxp3^+^) and tolerogenic dendritic cells (DCs) important in the suppression of immune responses. As an inhibitor of interleukin‐2 (IL‐2) signaling, sirolimus (SRL) blocks the proliferation of alloreactive T cells but facilitates the generation of Tregs, tolerogenic DCs, and a regulatory cytokine environment in vitro.^93^, ^94^ In contrast, CNIs block T cell receptor signal transduction and IL‐2 transcription, both inhibiting Treg generation.^95^, ^96^, ^97^ It has been previously demonstrated that CNI to SRL conversion increases systemic Tregs, regulatory DCs, and immunoregulatory proteogenomic signatures in liver transplant recipients, suggesting that it may facilitate immunosuppression minimization or withdrawal.^98^ Recently, a prospective trial of SRL monotherapy withdrawal was performed in non‐immune, non‐viremic LT recipients >3 years post‐LT.^99^ This study is the first to evaluate immunosuppression withdrawal directly from mTOR‐I therapy in LT recipients and achieved >50% operational tolerance. It would be expected that pre‐weaning blood/graft gene expression and PBMC profiling will be investigated as useful predictors of successful mTOR‐I therapy withdrawal.

## 9 LIVER TRANSPLANT DONOR

### 9.1 Donation after circulatory death (DCD)

For increasing the donor pool for orthotopic LT (OLT), the use of donation after circulatory death (DCD) donors is increasing. Although outcomes following DCD LT are worse than for donation after brainstem death (DBD) LT, it is uncertain whether a recipient should accept a “poorer quality” DCD organ or wait longer for a “better” DBD organ. Taylor et al^100^ reported the outcome after 953 DCD LTs in the UK registry. There was a survival advantage in accepting a DCD offer rather than waiting for a “better” DBD liver. Several concerns have been raised regarding the use of DCD liver. Jimenez‐Romero et al^101^ reported single‐center experiences of DCD LT. In the cohort, the recovery ratio of cannulated donors was 29.3%, and 75 livers were accepted for OLT. Although the rate of primary nonfunction and biliary complications were significantly higher in DCD recipients than DBD recipients, patient survival in recipients of DCD and DBD livers was similar. Narvaez et al^102^ demonstrated that pre‐mortem heparin administration status was not associated with liver discard but was associated with worse LT graft survival compared to heparin‐treated livers in US registry data including 5495 DCD organ recoveries. In addition, the study, which used data from the UK Transplant Registry, suggested a negative impact of prolonged hepatectomy time (HT), which means the time from aortic perfusion to end of hepatectomy, on outcomes on DCD LT.^103^ To be more specific, HT longer than 60 minutes was pointed out as an important factor for graft survival together with donor age of older than 45 years, CIT longer than 8 hours, and a recipient's previous abdominal surgery.

### 9.2 Machine perfusion

Recently, with attempts to expand the donor pool, attention has been focused on machine perfusion (MP) for marginal organs. MP is an emerging technology as a tool to assess graft viability and as a platform for graft intervention and modification.^104^ Active metabolism during MP facilitates targeted interventions for pretransplant graft treatment and modification to optimize preservation and maximize utilization. For livers, two main perfusion approaches are currently debated in the clinic: (a) perfusion with blood or alternative oxygen carriers at physiologic normothermic or subnormothermic conditions; or (b) perfusion with cooled oxygenated artificial fluids.^105^ Several signs of progress on MP have been reported in Table 5.^106^, ^107^, ^108^, ^109^, ^110^, ^111^, ^112^ The University of Oxford group designed a randomized, controlled study to test the potential of normothermic MP (NMP).^106^ Three hundred and thirty‐four livers offered for transplantation to eight European Centers were randomized to either conventional static cold storage (SCS) preservation or NMP. Median peak serum aspartate transaminase (AST), the primary endpoint of this study, was reduced by 49.4% in the NMP group when compared with the SCS group despite NMP livers having had longer functional warm ischemic times, longer overall preservation times, and fewer organ discards. The greatest benefit in a reduction in AST levels was observed in the DCD livers. The odds of NMP livers developing early allograft dysfunction were 74% lower compared with the SCS arm. Strikingly, organ discard rates were 24.1% for the SCS group compared to 11.7% for the NMP group, resulting in 20% more transplants performed in the NMP arm. Currently, several modified protocols, which include hypothermic or sequential protocol, have been reported. Machine perfusion of the liver has the potential to mitigate ischemic reperfusion injury via a shortening ischemic period of the livers or the reconditioning of their bioenergetic status.

**Table 5:** Clinical studies of machine perfusion reported in 2018/2019

Author	Center	Year	Timing of MP	RCT	Graft type	Endpoint	Clinical significance
Normothermic machine perfusion (NMP)							
Nasralla D^106^	UK	2018	Preserved MP	Yes	DBD/DCD	Peak AST, allograft dysfunction, graft use	Yes
Ghinolfi D^107^	Italy	2019	Post SCS MP	Yes	DBD, elderly	Graft and patient survival, IRI and biliary complications	Partially yes
Hypothermic machine perfusion (HMP)							
van Rijn R^108^	Netherlands	2018	Post SCS MP	No	DCD	IRI of bile duct	Yes
Schlegel A^109^	Switzerland	2019	Post SCS MP	No	DCD	Complication, patient survival, and graft loss	Yes
Muller X^110^	Switzerland	2019	Post SCS MP	No	DBD/DCD	Mitochondrial injury, allograft dysfunction, and early graft loss	Yes
Sequential							
van Leeuwen OB^111^	Netherlands	2019	Post SCS MP	No	DCD	Graft and patient survival, primary nonfunction, and cholangiopathy	Yes
de Vries Y^112^	Netherlands	2019	Post SCS MP	No	DCD	Graft survival	Yes

Abbreviations: AST, aspartate aminotransferase; DBD, donation after brain death; DCD, donation after circulatory death; IRI, ischemia‐reperfusion injury; SCS, static cold storage.

### 9.3 Viral exposed organ

The impact of hepatitis‐virus‐positive liver grafts, including hepatitis B and C, on survival and the risk of de novo hepatitis infection after LT remain controversial. Hepatitis‐virus‐negative patients on the LT waiting list may benefit from accepting virus‐exposed organs with preemptive treatment. Wong et al^113^ reported comparable perioperative and long‐term outcomes of hepatitis B core antibody (anti‐HBc) positive grafts after LT with antiviral monotherapy prophylaxis by analyzing 964 DBDLTs including cases with 416 anti‐HBc positive grafts. De novo HBV infection was observed in 4.7% (3/64) of HBs Ag‐negative recipients who received HBc positive liver with lamivudine, but no de novo infection was observed with entecavir prophylaxis (0/44). Furthermore, Lee et al^114^ reported that HBsAg‐positive deceased liver grafts worked well in HBsAg‐positive recipients with minimal viral activity under the treatment of combined antiviral nucleoside and nucleotide analogs. The use of HBsAg‐positive deceased grafts may be feasible for HBsAg‐positive patients and can increase the donor pool to rescue dying patients. HCV‐positive livers for LT have been considered for transplant in the era of DAA therapy.^115^ Several studies about transplanting HCV‐positive livers into HCV‐negative recipients with antiviral treatment were reported.^116^, ^117^, ^118^, ^119^, ^120^ However, in spite of encouraging initial outcomes, HCV positive to negative donation still must be taken only in the context of enhanced patient education and specific informed consent ideally within IRB‐approved protocols as recommended by the American Society of Transplantation.^121^

### 9.4 Living donor

Smaller surgical incisions have recently been innovated in living donor liver procurement. The Japanese nationwide survey reported that there were no significant differences in major complications between the standard incision and smaller incision, including laparoscopic approach.^122^ Park et al^123^ reported the outcome of the initial 91 cases in pure‐laparoscopic living‐donor right hepatectomy (LLDRH) procedure for LDLT. The incidence of major complication tends to be higher in the LLDRH group than the open laparotomy group but was not statistically significant in the propensity‐matched analysis. Further studies and technical improvement are needed to standardize the pure laparoscopic procedures, but smaller incision seems to have been adapted more frequently and be feasible in high‐volume centers. As another interesting topic, remote ischemic preconditioning (RIPC), which develops resistance for liver ischemia in living donors by transient ischemia and reperfusion of the arm, has attracted attention to avoid the harmful effects of ischemic reperfusion injury that is associated with graft dysfunction after LT. Veighey et al^124^ reported potential benefits of RIPC for post‐transplant liver function in recipients after LDLT based on the observation that early and maximum AST levels were significantly lower in the RIPC group (n = 75) compared to control group (n = 73) in the randomized clinical trial.

## 10 CONCLUSION

Liver transplantation has become a prevalent therapeutic option for a wide range of end‐stage liver diseases. Until recently, the largest proportion of LT in adults was performed in patients with HCV‐related cirrhosis. The availability of safe and effective DAAs to cure HCV infection in almost all patients regardless of the HCV genotype is currently reducing the need for LT. In contrast, NASH correspondingly escalates as an indication for LT. While facing such prominent alterations, there are serious challenges that are being studied steadily in the field of LT, i.e., the limited supply of donor organs, the indication criteria for patients with HCC, the appearance of DSA in sera, and the need for chronic immunosuppression, which represent the obstacles to the greater application and durable success of LT. In this article, we reviewed the current advances of those issues in LT.

## DISCLOSURE

Funding: This work was supported in part by JSPS KAKENHI Grant Numbers JP17K10669, JP18H05385, JP19H01057, and AMED under Grant Number 19fk021005h0001, 19ek0510029h0001. The founders had no role in study design, data collection and analysis, decision to publish, or preparation of the manuscript.

Conflict of Interest: The authors declare that they have no competing interests.
